# Relationship between foot morphology, muscle strength, and physical performance test in women aged 65 years and older: a cross-sectional study

**DOI:** 10.1186/s12891-022-05962-x

**Published:** 2022-11-18

**Authors:** Mieko Yokozuka, Kanako Okazaki, Masayuki Hoshi

**Affiliations:** Department of Physical Therapy, Fukushima Medical University School of Health Sciences, 10-6 Sakae-machi, Fukushima City, Fukushima, 960-8516 Japan

**Keywords:** Foot morphology, Muscle strength, Hallux valgus, Physical performance, Community-dwelling older individuals

## Abstract

**Background:**

Hallux valgus is a foot deformity that may affect gait, thus increasing the risk of falls among older people. We investigated the relationship between foot morphology, muscle strength, and physical performance.

**Methods:**

In this study, community-dwelling older people aged ≥65 years were included. A three-dimensional footprint automatic measurement apparatus was used to measure the hallux valgus angle, arch height ratio, and heel-floor angle. Furthermore, the toe flexor strength and ankle plantar flexion strength were measured. Physical performance tests included the five-repetition sit-to-stand test, one-leg standing time, maximal step length, functional reach test, and 5-m fastest walking time (walking time). The relationship between the hallux valgus angle and foot morphology and muscle strength was examined. In addition, factors affecting physical performance testing were assessed. Two-group comparisons, correlation, and multiple comparisons were used for statistical analyses.

**Results:**

Of the 133 women (age 77.7 ± 6.2 years), 57 had hallux valgus and 76 had no hallux valgus. There was a significant difference in the arch height ratio and heel-floor angle between women with and without hallux valgus (*p* < 0.001). A correlation was found between the hallux valgus angle and the heel-floor angle (r = 0.468, *p* < 0.001) and arch height ratio (r = − 0.337, *p* < 0.001), respectively. Multiple regression analysis showed that the hallux valgus angle was related to functional reach (β = − 0.162, *p* = 0.042), and toe flexor strength was related to five-repetition sit-to-stand (β = − 0.182, *p* = 0.036), maximal step length (β = 0.328, *p* < 0.001), and walking time (β = − 0.219, *p* = 0.006).

**Conclusions:**

A relationship was found between the hallux valgus angle, arch height rate, and inward inclination angle of the calcaneus. Functional reach was predicted based on the hallux valgus angle, whereas the five-repetition sit-to-stand, maximal step length, and walking time were predicted based on toe flexor strength. Hallux valgus predicted not only the forefoot but also the foot morphology and was related to physical performance. From the perspective of motor function and fall prevention, efforts should be made to better understand and prevent the onset and progression of hallux valgus.

## Background

Pain and deformity of the feet and toes during walking have been reported to be associated with falls [1, 2]. Falls in older individuals are associated with a higher odds ratio of 1.95 for foot pain and 1.89 for hallux valgus [2]. The overall prevalence of definite radiographic diagnosis of hallux valgus was 22.8% in community-dwelling individuals aged ≥65 years, and 11.6 and 41.1% in older men and women, respectively [3]. In addition, 42% of women aged > 80 years have hallux valgus, and the frequency is higher in women than in men, indicating the need for foot care [4]. Although the prevalence of hallux valgus in women is high, it is believed that several older people do not seek treatment because they do not experience pain.

Hallux valgus is characterized not only by a deformity of the hallux but also by an effect on the arch of the foot, causing a decrease in the medial longitudinal arch in flat feet [5, 6] and expansion of the transverse arch in splay feet [6, 7]. Furthermore, it affects the entire foot since the lateral foot pressure decreases during walking in hallux valgus, suggesting that the foot is in pronation due to a decrease in the arch height ratio [8]. Hallux valgus possibly affects not only the forefoot but also the entire foot. However, there are no reports measuring eversion of the foot in hallux valgus nor are there any reports on the relationship between the hallux valgus angle, arch height ratio, and rear foot morphology.

There have been several reports on the toes and motor function. A study of community-dwelling older people reported that hallux valgus and lesser toe deformities reduce toe flexor strength and increase the risk of falls [9]. It has been reported that the plantar flexion strength of the hallux and the range of motion of the ankle joint are essential for balance and functional ability [10].

Hallux valgus may not merely be restricted to a deformity of the hallux but also extend to the overall morphology of the foot, which in turn affects motor function, although this aspect has not been examined in prior studies. Therefore, this study aimed to measure foot morphology using a three-dimensional (3D) foot measurement device in community-dwelling older people and clarify the relationship between foot morphology and motor functions that may involve the toes.

## Methods

### Participants

The study was approved by the Ethics Committee of Fukushima Medical University (approval number: 2020–117). This cross-sectional study included participants who were recruited from ≥65-year-old residents near the measurement site of this study and who participated in self-management group exercise classes held near their homes. A document describing the study content was distributed before measurement. This study included individuals who agreed to participate and presented to the measurement site. The exclusion criteria for the analysis were as follows: [1] motor paralysis due to a history of cerebrovascular disease; [2] use of psychotropic medication or a history of psychiatric consultation; and [3] walking aid required for indoor mobility. The proportion of older Japanese community-dwelling people who developed hallux valgus was 15.5 and 36.9% among men and women, respectively [3]. Suppose the effect size is 0.5, α-value is 0.05, and power (1-β) is 0.8 for the unpaired t-test, the required sample size would be 38 individuals with hallux valgus for a group of 236 men and 51 individuals with hallux valgus for a group of 138 women, to ensure adequate statistical power (G*Power 3.1.9.7).

A flowchart of the selection of participants for the analysis is presented in Table 1. Overall, 187 participants were included in this study. Notably, three participants had a history of cerebrovascular disease, two participants used psychotropic medication or had a history of psychiatric consultation, one participant required a walking aid for indoor mobility, and one participant had a measurement error. The number of participants available for analysis was 133 women and 47 men. Consequently, the number of men was insufficient for this analysis.

**Table 1:** Flowchart of participants analyzed

Participants: 48 men, 139 women	
↓	
Exclusion criteria: motor paralysis due to a history of cerebrovascular disease *n* = 3 (1 man, 2 women) taking psychotropic medication or with a history of psychiatric consultation: *n* = 2 (2 women) requiring a walking aid for indoor mobility: *n* = 1 (1 woman) a measurement error: *n* = 1 (1 woman)	
↓	
Participants analyzed: 133 women (47 men were excluded from the analysis due to the small sample size)	

### Measurements

Measurements were performed by three physical therapists with experience in collecting data in the community. The following basic information was obtained from the participants: date of birth, height, weight, current and past medical history, medications, and presence of foot pain. Height and weight were recorded as self-reported values.

All measurements of foot morphology and motor function were performed barefoot. The foot morphology measurement is generally confirmed by radiography in medical institutions; however, since we targeted community-dwelling older people, we used a simple and noninvasive method. Foot morphology was measured using a 3D footprint automatic measurement apparatus (JMS-3100; Dream GP Inc., Osaka, Japan). This foot-scanning system uses a rail-type laser to measure the entire circumference of one foot, including the instep, heel, toes, and sole, at approximately 30,000 locations on each foot. First, in the standing position, markers with a diameter of 5 mm were attached to the navicular tuberosity, bottom of the calcaneal tuberosity, and enthesis of the Achilles tendon [11]. The posture for measurement was a static standing position with the feet a shoulder-width apart and equal weight applied to both feet. The participants placed one foot in the measuring device and performed two measurements on each side. A calibrated foot scanner was used. The accuracy of the foot-scanning system is ±1.0 mm for the foot length and ± 1.5 mm for the foot perimeter [12]. Foot morphology was measured using the following parameters. The hallux valgus angle was measured as the angle between the line connecting the first metatarsal head and the first proximal phalanx head, and the line connecting the first metatarsal head and the posterior medial malleolus. A hallux valgus angle of 20° or more on radiography, which is considered to indicate hallux valgus, corresponds to a hallux valgus angle of 16° determined from the contour of the footprint [13]. Therefore, we determined the presence or absence of hallux valgus according to a hallux valgus angle of ≥16° or < 16°, respectively. If the bilateral or unilateral hallux valgus angles were ≥ 16°, the side with the greater hallux valgus angle was analyzed. When the hallux valgus angle was < 16° on both sides, the side to be analyzed was selected equally using random numbers. The arch height ratio was calculated as the ratio of the floor-to-the-navicular-tuberosity height to foot length. The heel-floor angle (HFA) was measured as the angle between the line connecting the bottom of the calcaneal tuberosity and enthesis of the Achilles tendon and the vertical line from the floor surface. The line connecting the bottom of the calcaneal tuberosity and enthesis of the Achilles tendon was measured as positive (+) when the line sloped inward from the vertical line from the floor and the sole turned outward (eversion), and negative (−) when the line sloped outward and the sole turned inward (inversion). The first measurement was adopted; however, if the foot was moved during the measurement and could not be measured, the second measurement was adopted.

Toe flexor strength and ankle plantar flexion strength were measured as toe muscle strength. The following devices were used to measure the muscle strength of the feet and toes, which can be measured quantitatively and easily in the community. The toe flexor strength was measured using a toe grip dynamometer (T.K.K. 3364b; Takei Scientific Instruments, Niigata, Japan). The posture for measurement was a chair sitting position with the hip and knee joints at 90° flexion. To prevent supination of the ankle joint, it was fixed with a belt, and the force to grip the bar with the toes was measured [14]. The measurements were performed twice on each side. The ankle plantar flexor strength was measured using a hand-held dynamometer (μTasF-1; Anima Co., Tokyo, Japan). A study reported a method of measurement in a long-sitting position on the bed, where both intraclass correlation coefficient (ICC) values measuring the intra- and inter-rater reliability were over 0.9 [15], and this method was used as a reference. Because some people have difficulty assuming a long-sitting position with both feet together, only the lower limbs to be measured in the chair sitting position were assumed to be in the long-sitting position, with the knees extended and the soles of the feet placed against the wall at 0° of plantar flexion/dorsiflexion. A hand-held dynamometer was inserted between the wall and sole of the foot, and a sensor was placed proximal to the metatarsophalangeal joint to measure the isometric contraction of ankle plantar flexion. The participants were reminded not to push the hand-held dynamometer with the extensor muscles of the trunk, hip, and knee joints. The measurements were performed twice on each side. Toe flexor strength and ankle joint plantar flexion strength were calculated by dividing the maximum values of the right and left sides by the body weight.

The following five items were measured as performance tests in which the toes were considered involved. In the five-repetition sit-to-stand test (5R-STS), the starting position was a chair with the feet a shoulder-width apart, both arms crossed in front of the chest, and the time to repeat the five standing and sitting movements as quickly as possible was measured once using a stopwatch. A chair with a height of approximately 42 cm was used. A previous study reported that the ICC values of 5R-STS range from 0.64 to 0.96 [16]. The one-leg standing time was measured with a stopwatch, which measured the length of time for which the participant could stand with one leg raised above the floor by a few centimeters. The maximum time allowed for each test was 60 s, which was performed twice on each side. The maximum value on each side was used for analysis. ICC_2,1_ indicated excellent relative reliability for single-leg-stance time (ICC = 0.86) [17]. The maximal step length (MSL) was determined by measuring the distance from a standing position with the feet shoulder-width apart to a standing position with the foot contralateral to the target foot stepped forward as far as possible, followed by placing the target foot standing in front of it. The measurements were performed twice on each side. The MSL showed the best ICCs [18, 19]. The functional reach test (FR) was performed in a standing position with the feet a shoulder-width apart, arms flexed 90° at the shoulder joint, and the distance from this position to the position where the hands were reached as far forward as possible was measured with a measuring tape. A study has shown acceptable reliability of the FR [20]. The measurements were performed twice. The MST and FR were calculated by dividing the maximum value by the height. The 5-m fastest walking time (walking time) was measured by walking 11 m as fast as possible, which consisted of a 5-m walking path plus a 3-m front and rear approach, and the time spent walking the 5-m walking path was measured once with a stopwatch. The correlation coefficients between the two trials at speeds “as fast as possible” were 0.93, and their reproducibility has been confirmed in a previous study [21].

To reduce the time required for measurement, the measurement was performed by deciding the line of flow. Furthermore, the measurements were taken in the same order to ensure consistent conditions. The measurements were performed with explanations included at any time, and practice and rest were performed as necessary.

### Statistical analysis

The Shapiro–Wilk test was used to test the normality of the data. The differences in age, weight, body mass index (BMI), arch height ratio, toe flexor strength, and MSL between the two groups were analyzed using the unpaired t-test. The other variables were tested using the Mann-Whitney U test. Spearman’s correlation coefficient was performed to determine the relationship between the participants’ characteristics and foot morphology, muscle strength, and physical performance. The physical performance test was the dependent variable. Additionally, age using forced entry, height, weight, hallux valgus angle, toe flexor strength, and ankle plantar flexion strength using stepwise were the explanatory variables analyzed in hierarchical multiple regression analyses. Statistical analyses were performed using IBM SPSS for Windows (version 28.0; IBM Corp., Armonk, NY, USA), and the significance level was set at a *p*-value of < 5%.

## Results

Overall, 133 women (age 77.7 ± 6.2 years, height 151.4 ± 5.8 cm, weight 50.6 ± 6.7 kg, BMI 22.1 ± 2.8 kg/m^2^) were included in the analysis. Participants’ characteristics are presented in Table 2. Additionally, 57 women had at least one hallux valgus and 76 had no hallux valgus.

**Table 2:** Characteristics of the participants

	HV, *n* = 57	No HV, *n* = 76	*p*-value
Age: years	77.7 ± 6.4	77.7 ± 6.0	0.955
Height: cm	151.0 ± 5.4	151.7 ± 6.1	0.324
Weight: kg	50.3 ± 6.1	50.9 ± 7.1	0.581
BMI: kg/m^2^	22.1 ± 2.6	22.2 ± 3.0	0.849
Presence of foot pain: n	1	2	1.000

*Abbreviations*: *HV* hallux valgus, *BMI* body mass index

Foot morphology and motor function of both groups are shown in Table 3. With hallux valgus, the arch height ratio was low (*p* < 0.001), the HFA became everted, and the calcaneus was tilted inward (*p* < 0.001). There was no significant difference in toe flexor strength between the two groups. In addition, the time to 5R-STS and the FR distance were shorter in the hallux valgus group (*p* = 0.032 and *p* = 0.014, respectively).

**Table 3:** Comparison of foot morphology and motor function in participants with and without hallux valgus

	HV, *n* = 57	No HV, *n* = 76	*p*-value
HV angle: degree	24.4 ± 6.6 (16.5 – 41.4)	8.7 ± 4.0 (0 – 15.4)	< 0.001
Arch height ratio: cm/cm*100	15.4 ± 2.4	17.3 ± 2.6	< 0.001
HFA: degree	3.4 ± 3.5	1.1 ± 2.6	< 0.001
Toe flexor strength: kgf/wb*100	20.1 ± 9.0	20.4 ± 8.2	0.860
Ankle plantar flexion strength: kgf/wb*100	17.5 ± 6.7	19.7 ± 7.0	0.016
5R-STS: seconds	9.0 ± 3.0	9.8 ± 2.9	0.032
OLST: seconds	33.4 ± 22.8	34.8 ± 23.9	0.462
MSL: cm/cm *100	63.0 ± 8.8	62.1 ± 8.7	0.566
FR: cm/cm *100	19.4 ± 3.4	20.8 ± 3.3	0.014
Walking time: seconds	2.9 ± 0.6	3.1 ± 0.7	0.206

*Abbreviations*: *HV* hallux valgus, *HFA* heel-floor angle, *5R-STS* five-repetition sit-to-stand, *OLST* one-leg standing time, *MSL* maximal step length, *FR* functional reach test

The relationships between the participants’ physical characteristics and their foot morphology and motor function are presented in Table 4. Furthermore, a correlation between age and physical performance was observed.

**Table 4:** Correlation between physical characteristics, foot morphology, and motor function

	HV angle	Arch height rate	HFA	Toe flexor strength	Ankle plantar flexion strength	5R-STS	OLST	MSL	FR	Walking time
Age	−0.024	−0.064	0.095	−.182^*^	− 0.007	.206^*^	−.474^**^	−.250^**^	−.365^**^	.426^**^
Height	0.022	0.112	0.063	.176^*^	0.105	−0.074	.243^**^	0.096	0.158	−.281^**^
Weight	−0.008	−0.067	− 0.062	−0.044	− 0.050	−0.074	− 0.017	−0.059	0.056	−0.053

*p < 0.05, **p < 0.01

*Abbreviations*: *HV* hallux valgus, *HFA* heel-floor angle, *5R-STS* five-repetition sit-to-stand, *OLST* one-leg standing time, *MSL* maximal step length, *FR* functional reach test

Units: Age: years, Height: cm, Weight: kg, HV angle: degree, Arch height rate: cm/cm*100, HFA: degree, Toe flexor strength: kgf/wb*100, Ankle plantar flexion strength: kgf/wb*100, 5R-STS: seconds, OLST: seconds, MSL: cm/cm *100, FR: cm/cm *100, Walking time: seconds

The relationship between foot morphology and motor function is presented in Table 5. As the hallux valgus angle increased, the arch height ratio decreased (r = − 0.337, *p* < 0.001) and the HFA pronated (r = 0.468, *p* < 0.001). There was a negative correlation between the hallux valgus angle and FR (r = − 0.218, *p* = 0.012). The relationships between toe flexor strength and 5R-STS (r = − 0.236, *p* = 0.007), OLST (r = 0.247, *p* = 0.004), MSL (r = 0.361, *p* < 0.001), FR (r = 0.229, *p* = 0.008), and walking time (r = − 0.337, *p* < 0.001) were found to be significant.

**Table 5:** Correlation between foot morphology and motor function

	HV angle	Arch height rate	HFA	Toe flexor strength	Ankle plantar flexion strength	5R-STS	OLST	MSL	FR	Walking time
HV angle	1.000	−.337^**^	.468^**^	−0.024	−.188^*^	− 0.170	− 0.068	0.046	−.218^*^	− 0.093
Arch height rate		1.000	−.338^**^	0.053	.288^**^	.173^*^	0.095	−0.098	0.051	0.056
HFA			1.000	0.076	−0.040	− 0.168	− 0.041	.184^*^	0.000	−0.105
Toe flexor strength				1.000	0.153	−.236^**^	.247^**^	.361^**^	.229^**^	−.337^**^
Ankle plantar flexion strength					1.000	0.050	0.023	0.087	.220^*^	−0.119

*p < 0.05, **p < 0.01.

*Abbreviations*: *HV* hallux valgus, *HFA* heel-floor angle, *5R-STS* five-repetition sit-to-stand, *OLST* one-leg standing time, *MSL* maximal step length, *FR* functional reach test

Units: HV angle: degree, Arch height rate: cm/cm*100, HFA: degree, Toe flexor strength: kgf/wb*100, Ankle plantar flexion strength: kgf/wb*100, 5R-STS: seconds, OLST: seconds, MSL: cm/cm *100, FR: cm/cm *100, Walking time: seconds

The results of the multivariate analysis of the physical performance tests are presented in Table 6. The hallux valgus angle was associated with FR (β = − 0.162, *p* = 0.042). Toe flexor strength was associated with 5R-STS (β = − 0.182, *p* = 0.036), MSL (β = 0.328, *p* < 0.001), and walking time (β = − 0.219, *p* = 0.006).

**Table 6:** Multivariate analysis of physical performance tests

Physical performance test	Explanatory variables	R2	Adjusted R2	Durbin-Watson ratio	F-value	B	SE	Standardized β	t	*p*-value	VIF
5R-STS	Age	0.067	0.053	2.005	4.677	0.074	0.041	0.155	1.796	0.075	1.032
	Toe flexor strength					−0.063	0.030	− 0.182	−2.119	0.036	1.032
OLST	Age	0.281	0.264	1.975	16.818	−1.798	0.288	−0.477	−6.249	< 0.001	1.046
	Weight					−0.840	0.279	−0.241	−3.004	0.003	1.156
	Height					0.807	0.322	0.200	2.506	0.013	1.146
MSL	Age	0.169	0.157	2.312	13.263	−0.278	0.114	−0.197	−2.429	0.017	1.032
	Toe flexor strength					0.336	0.083	0.328	4.041	< 0.001	1.032
FR	Age	0.217	0.199	1.895	11.910	−0.204	0.043	−0.367	−4.712	< 0.001	1.001
	Ankle plantar flexion strength					0.110	0.039	0.222	2.812	0.006	1.024
	HV angle					−0.059	0.029	−0.162	−2.055	0.042	1.024
Walking time	Age	0.249	0.232	2.047	14.269	0.036	0.008	0.337	4.302	< 0.001	1.052
	Toe flexor strength					−0.017	0.006	−0.219	−2.803	0.006	1.050
	Height					−0.020	0.009	−0.176	−2.253	0.026	1.044

*Abbreviations*: *5R-STS* five-repetition sit-to-stand, *OLST* one-leg standing time, *MSL* maximal step length, *FR* functional reach test, *HV* hallux valgus, *SE* standard error, *VIF* variance inflation factor

Units: 5R-STS: seconds, OLST: seconds, MSL: cm/cm *100, FR: cm/cm *100, Walking time: seconds

## Discussion

The purpose of this study was to investigate the effect of hallux valgus on foot morphology. In our study of women aged ≥65 years, hallux valgus was associated with a low arch height ratio and the HFA was associated with eversion. Furthermore, there was a correlation between the hallux valgus angle, arch height ratio, and HFA. It has been reported that hallux valgus is not only accompanied by an increase in the hallux valgus angle but also by the turning of the first metatarsal [22]. The relationship between hallux valgus and the arch height ratio, which measures the height of the navicular bone from the floor, has also been reported [6]. Deformities of the toes affect not only the forefoot but also the midfoot. Moreover, regarding plantar pressure loading during gait, it has been reported that a foot with hallux valgus is in pronation because of the position of the center of pressure excursion relative to the foot width [8]. Hallux valgus may affect the sole and foot; however, no reports have measured calcaneal tilt. In this study, the calcaneus was inclined medially in the static standing position, and it was clear that hallux valgus affected the morphology of the entire foot, including the rearfoot.

Furthermore, we examined whether such changes in foot morphology can predict motor function, including muscle strength. In this study of women aged ≥65 years, we found no difference in toe flexor strength between women with and without hallux valgus and no relationship between hallux valgus angle and toe flexor strength. In a study of women aged 19.6 ± 1.3 years, toe flexor strength significantly decreased in women with hallux valgus [23]. Toe flexor strength has been reported to be affected by age and sex [24]. In addition, toe flexor strength in women aged ≥80 years has been reported to be positively correlated with the average number of steps taken per day in daily life [25]. The mean age of the participants in this study was 77.7 ± 6.2 years, which was a wider range of participants’ ages compared to the aforementioned study of young adults. It is believed that the effect of the age range and the difference in activity of the participants in this study caused the absence of any observed relationship between hallux valgus and toe flexor strength.

This study found a correlation between age and all physical performance tests; thus, age was forcibly entered. While height, weight, hallux valgus angle, and muscle strength were entered in a stepwise manner, the factors predicting physical performance were analyzed by hierarchical multiple regression analysis. Additionally, hallux valgus angle and toe flexor strength predicted physical performance, even when age was considered. The presence of hallux valgus significantly decreased FR, indicating the influence of the hallux valgus angle on FR. In foot plantar pressure during walking with hallux valgus, hallux peak pressure [26], contact time, and maximum forces [27] were reduced, and hallux dysfunction was reported. When the center of gravity is shifted forward in the FR, the pressure on the toe with hallux valgus may decrease. It is possible that when the center of gravity is shifted forward in the FR, the tip of the hallux cannot be sufficiently loaded owing to hallux valgus.

Toe flexor strength predicted the 5R-STS, MSL, and walking time test results. Several studies have shown a relationship between toe flexor strength and physical performance. It has been reported that the timed-up-and-go test, a performance test, is associated with toe flexor strength [28]. In addition, a relationship between toe flexor strength and walking speed, single-leg support time, and stride length has been reported [29]. Further, it has been reported that toe flexor strength is correlated with the cross-sectional area of the flexor hallucis brevis, flexor digitorum brevis, and abductor hallucis muscles [30]. These intrinsic muscles are active during the push-off phase of the gait cycle [31]. It has been recognized that toe flexor strength is important in walking and movements that include this element. It is unclear whether it is possible to improve toe flexor strength and motor functions, such as 5R-STS, MSL, and walking time, by inserting a sole plate that supports the arch and calcaneus. In the future, examining the effect of sole plate insertion on the function of feet with hallux valgus is necessary.

The limitations of this study are as follows. First, there was a wide variation in the participants’ ages. It is necessary to examine whether the effect of the hallux valgus angle on toe flexor strength is determined by age. However, our study could not clarify this association because the study sample was too small to be classified by age. Second, toe flexor strength was affected by activities of daily living, but the effect was unclear because we did not collect information on the amount of activity.

## Conclusion

This study found an association between increased hallux valgus angle, decreased arch height ratio, and medial inclination of the calcaneus. However, the causal relationship could not be ascertained. FR was predicted by the hallux valgus angle, while 5R-STS, MSL, and walking time were predicted by toe flexor strength. Therefore, hallux valgus causes forefoot deformities and predicts foot morphology and motor function. Preventing the onset and deterioration of hallux valgus will help to reduce balance-related falls in the older population.

## Data Availability

The datasets generated and analyzed during the current study are not publicly available because we did not obtain the consent of the participants to publish the individual data. However, the datasets are available from the corresponding author upon reasonable request.

## Abbreviations

HV: Hallux valgus
HFA: Heel-floor angle
5R-STS: Five-repetition sit-to-stand test
OLST: One-leg standing time
MSL: Maximal step length
FR: Functional reach test
